# Integration of Multi-Omics Data To Understand the Multifaceted Role of RAMP1 across Different Cancer Types

**DOI:** 10.1007/s12013-026-02021-3

**Published:** 2026-04-21

**Authors:** Gabrielle da Costa Cabral, Letícia Almeida Rodrigues, Júlia de Almeida Cabral Candiago, João Felipe Fernandes Ferreira Procópio, Júlia Costa Cerqueira Silva, Maria Eduarda Barbosa de Lima, Ana Stella Gisela Ellery Costa Lima, José Valmir Falcão da Costa Júnior, David Abner Santos de Araújo, João Paulo Emiliano da Silva, Sofia Amancio de Almeida Oliveira, Rafael de Oliveira Calaça Farias, Leonardo Prudêncio Coutinho de Almeida, Salomão Belfort Sparapan de Melo, José Emerson Xavier, Sandra Taveiros de Araújo, Raimundo Rodrigues de França-Júnior, Maria Amélia dos Santos Lemos Gurgel, Carlos Alberto de Carvalho Fraga

**Affiliations:** 1 Centro de Ciências Médicas, Universidade Federal de Alagoas, Campus Arapiraca, Av. Manoel Severino Barbosa, Bom Sucesso, Arapiraca, CEP 57309-005 AL Brazil; 2 CMOL - Computational and Molecular Laboratory, Av. Manoel Severino Barbosa, Bom Sucesso, Arapiraca, CEP 57309-005 AL Brazil

**Keywords:** Receptors, g Protein-Coupled, Receptor Activity-Modifying protein 1, Medical oncology, Neoplasms, Multi-omics, Calcitonin Receptor-Like receptor

## Abstract

Receptor Activity-Modifying Protein 1 (RAMP1) is a Calcitonin Receptor-Like Receptor (CALCRL)-associated protein with significant relevance in oncogenesis and tumor progression. It acts as a context-dependent allosteric modulator of GPCRs. This integrative review synthesizes evidence from multiple omics layers—transcriptomics, proteomics, epigenomics, and pan-cancer datasets—to clarify the role of RAMP1, its signaling pathways, and its relationship with the tumor microenvironment. Multi-omics integration highlighted several key findings: transcriptomic and regulatory signatures frequently associate RAMP1 overexpression with epithelial–mesenchymal transition; RAMP1 is associated with the coordinated activation of GPCR-dependent pro-tumorigenic pathways; and the CGRP/RAMP1 axis is associated with CD8^+^ T-cell exhaustion and potential resistance to immune checkpoint inhibitors. Pan-cancer analyses revealed marked expression heterogeneity. RAMP1 is strongly overexpressed in hepatocellular carcinoma (LIHC) and downregulated in kidney renal clear cell carcinoma (KIRC), reinforcing the importance of context-specific interpretation. It also emerges as an unfavorable prognostic biomarker in osteosarcoma, rectal adenocarcinoma, prostate cancer, and Ewing sarcoma, suggesting its potential value as a strategic therapeutic target. Future studies should prioritize spatial transcriptomics and the pharmacological modulation of the CGRP/RAMP1 axis to refine precision oncology strategies.

## Introduction

Recent evidence from multi-omics analyses has highlighted the emerging role of receptor activity-modifying protein 1 (RAMP1) in tumor immunity. Studies show that RAMP1 expression correlates with immune checkpoint genes (ICGs) and with the presence of tumor-infiltrating lymphocytes (TILs), in addition to influencing immune and inflammatory responses within the tumor microenvironment (TME) [1]. As an essential component of the calcitonin gene-related peptide (CGRP) receptor, RAMP1 may also impair the cytotoxic function of CD8⁺ T cells, thereby potentially reducing tumor cell elimination. These findings suggest that RAMP1 represents not only a promising prognostic biomarker but also a potential therapeutic target that could help in enhancing immunotherapy outcomes [2]. To better understand its physiological role and its relationship with tumor tissues, it is necessary to examine its structure and the signaling pathways in which it is involved.

### Overview of RAMP1 and the CGRP Pathway

RAMP1 belongs to a family of three single-pass transmembrane proteins (RAMP1, RAMP2, and RAMP3) [3]. These proteins are widely expressed in human tissues and are unique to vertebrates [4]. RAMP1 contains a structured extracellular N-terminal domain of approximately 90–100 amino acids and a short cytoplasmic C-terminal tail [4]. The fundamental function of these proteins, including RAMP1, is to interact with G protein-coupled receptors (GPCRs) and modulate their activity. In this context, they act as allosteric regulators of GPCR function [4].

In parallel, GPCRs play a central role in transducing extracellular signals into specific intracellular responses. At the cellular level, they function as molecular sensors capable of recognizing a wide range of ligands, including hormones, neurotransmitters, chemokines, and metabolites, and can undergo conformational changes that lead to the activation of heterotrimeric G proteins [5]. Canonically, GPCR activation results in the modulation of second messengers, such as cyclic AMP (cAMP), which subsequently triggers intracellular signaling cascades that regulate processes including proliferation, differentiation, migration, cell survival, and apoptosis [5].

Within the tumor microenvironment, GPCR-mediated responses are driven by multiple ligands, including chemokines, bioactive lipids, metabolites, and hormones. This signaling enables neoplastic cells to modulate their behavior in response to inflammatory, metabolic, and immune cues [5]. GPCR activation has been shown to promote stimulation of pro-growth pathways, such as MAPK/ERK and PI3K/AKT, thereby potentially favoring uncontrolled cellular growth and tumor proliferation. This signaling can be maintained through receptor overexpression, autocrine ligand production, or paracrine interactions with cells of the microenvironment, resulting in persistent signaling circuits that drive cancer progression [5].

Current research on the oncogenic effects of GPCRs has highlighted the importance of intracellular adaptor proteins, particularly β-arrestins (ARRB1 and ARRB2). Recent studies indicate that these proteins act as regulators of GPCR desensitization and internalization while also emerging as G protein–independent signaling platforms capable of redirecting signaling flux toward pathways associated with tumor development [6].

In the context of hepatocellular carcinoma, this ability to modulate signaling flow is particularly relevant, as β-arrestins function as molecular integrators that connect inflammatory, metabolic, and viral stimuli to sustained activation of classical oncogenic pathways, such as PI3K/AKT and MAPK, as well as parallel pathways including GSK-3β and mTOR [6]. Similarly, several studies suggest that RAMP1 expression is associated with the activation of these classical signaling pathways [7, 8], highlighting the need for a deeper discussion of the mechanisms underlying GPCR signaling and their associated adaptor proteins.

### Evidence of RAMP1 Expression Across Tumor Tissues

To further characterize RAMP1 expression, pan-cancer gene expression analyses have demonstrated that RAMP1 displays distinct expression patterns when comparing normal and tumor tissues, with additional variability across cancer types. Tumors such as hepatocellular carcinoma, lung cancer, breast cancer, thyroid cancer, and prostate cancer exhibit increased RAMP1 expression [1]. Moreover, high RAMP1 expression has been associated with an increased risk of metastasis in patients with osteosarcoma [9], supporting its potential use as both a diagnostic biomarker and a prognostic parameter. Functional evidence further suggests that cancer cells may leverage the RAMP1/CGRP pathway to support tumor progression by promoting cell proliferation, angiogenesis, invasion, and immunosuppression.

### Rationale for the Use of Multi-Omics Approaches

Multi-omics approaches refer to the integration of diverse omics technologies to enable interactive analyses of genomic, transcriptomic, epigenomic, proteomic, and metabolomic data [10]. Cancer is a fundamentally heterogeneous disease, characterized by substantial variability in cellular architecture, growth dynamics, and genetic material. As a result, studying cancer through a single omics layer is insufficient to fully capture its complexity. Multi-omics strategies provide the comprehensive perspective required to address this multifaceted condition.

The relevance of this integrative approach is further supported by observations in ovarian carcinoma, which represents a heterogeneous group of tumors with distinct molecular profiles [10]. These tumors exhibit marked variation in gene expression patterns, genetic mutations, and epigenetic alterations.

### Aims and Scope of This Review

This review aims to comprehensively elucidate the complex role of RAMP1 in cancer by systematically integrating evidence from genomics, transcriptomics, proteomics, and epigenomics. We also discuss how RAMP1 influences tumor progression and immune modulation, and examine its potential as a diagnostic biomarker, prognostic parameter, and therapeutic target. By synthesizing multi-omic findings, this review provides a broad and updated perspective on the molecular mechanisms that govern RAMP1 function in oncogenesis and highlights opportunities for advancing precision immunotherapy.

## Protein Interactions and Signaling Mediated by RAMP1

### Interactome: Functional Partners of RAMP1 in CGRP Pathways and G Protein-Coupled Receptors

Within the broader landscape of GPCR signaling, the most prominent and best-characterized role of RAMP1 occurs within the CGRP pathway, in which CGRP is primarily secreted by sensory neurons [4]. RAMP1 functions as an essential co-receptor for the Calcitonin Receptor-Like Receptor (CALCRL), a seven-transmembrane G protein–coupled receptor [11]. The CALCRL/RAMP1 complex forms the canonical CGRP receptor (CGRPR) [4], and this interaction is required for the correct transport and expression of the receptor at the cell surface. RAMPs act as chaperones for GPCRs by assisting their exit from the endoplasmic reticulum, ensuring proper folding and functional assembly [4]. They also influence ligand recognition by modulating receptor specificity toward peptide ligands [4, 11].

Binding of CGRP to the CALCRL/RAMP1 receptor complex triggers an increase in intracellular cAMP and subsequent activation of protein kinase A (PKA), initiating downstream signaling cascades [11]. CGRP participates in physiological functions such as blood pressure regulation, neurotransmission and wound healing, and its dysregulation has been linked to pathological conditions including migraine and cancer development, particularly through its pro-angiogenic properties [11].

**Fig. 1:**
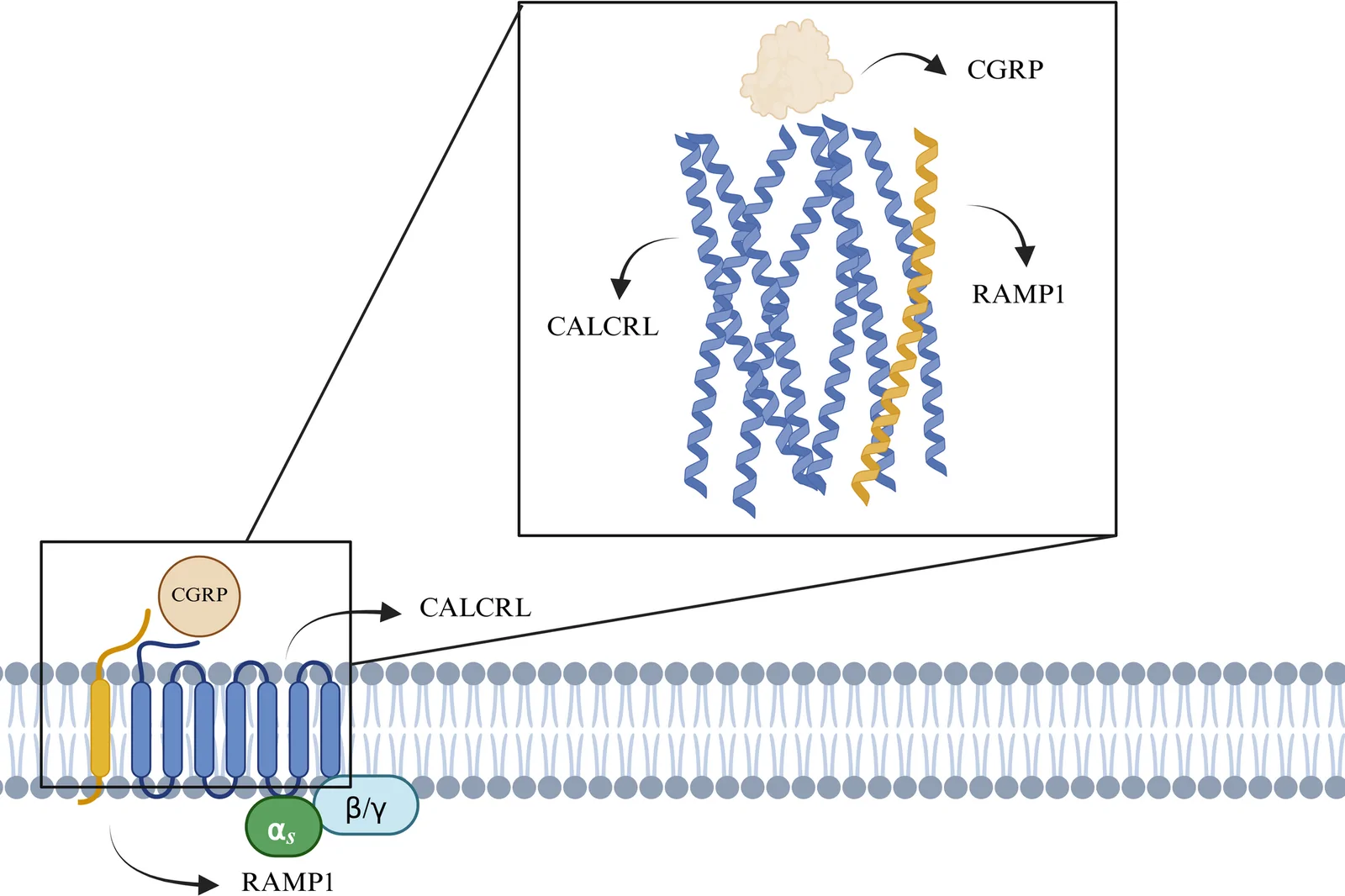
Schematic representation of the CGRP/CALCRL/RAMP1 receptor–ligand complex, illustrating the interactions involved in for CGRPR activation. The diagram illustrates the architecture of the functional CGRP receptor (CGRPR), which is a heterocomplex formed by the Calcitonin Receptor-Like Receptor (CALCRL), a G protein–coupled receptor (GPCR), and its associated coreceptor, Receptor Activity-Modifying Protein 1 (RAMP1). The transmembrane portion of RAMP1 interacts with CALCRL, positioning itself near Transmembrane Helix 4 and Transmembrane Helix 2 (ECL2), as shown in the image. This RAMP1/CALCRL interaction is essential, as RAMP1 acts as an allosteric modulator, stabilizing the conformations of the extracellular domain of CALCRL and of the ECL2 loop. This stabilization plays a key role to create a favorable environment at Ligand Binding Site 2, supporting high-affinity recognition and subsequent binding of the peptide ligand CGRP. The binding of CGRP to the complex triggers intracellular signaling, such as the increase in cAMP

Cryo-EM studies have shown that RAMP1 establishes an extensive interface with CALCRL, involving approximately 23% of its molecular surface. Its transmembrane segment inserts between helices TM2, TM3 and TM4, whereas its extracellular domain interacts with both the CALCRL extracellular domain and loop ECL2, as illustrated in Fig. 1. This structural organization stabilizes the receptor and shapes the conformation required for ligand binding. In the active CGRP receptor, CALCRL and RAMP1 operate as a heterocomplex in which RAMP1 modulates the orientation of the extracellular domain and ECL2, allowing the formation of a functional binding pocket. Once accommodated in this site, CGRP triggers the canonical signaling pathway leading to a rise in cAMP [4].

Beyond its structural role, RAMP1 acts as an essential allosteric modulator of CGRP signaling. Structural analyses indicate minimal direct contact between RAMP1 and CGRP, suggesting that RAMP1 primarily stabilizes specific conformations of the CALCRL extracellular domain and ECL2. This stabilization enhances CGRP binding and promotes downstream cAMP-dependent signaling [4].

Another relevant complex involving RAMP1 is the CALCR/RAMP1 heterodimer, which forms the Amylin Receptor 1 (AMY1). This hybrid receptor shows high affinity for both amylin and CGRP, classifying it as a dual-ligand receptor with diverse physiological roles [4]. Amylin is a 37-amino-acid peptide hormone co-secreted with insulin by pancreatic β-cells, acting in the regulation of food intake and glucose metabolism [4].

In addition to these classical interactions, RAMP1 associates with a wide range of GPCRs from both Class B (Secretin family) and Class A (Rhodopsin family). Within Class B, interacting receptors include GIPR, GLP2R, PTH1R, PTH2R, VIPR1 and VIPR2, all of which participate in metabolic and hormonal regulation. Within Class A, RAMP1 interacts with numerous chemokine receptors—including ACKR1, CCR1, CCR3, CCR4, CCR6, CCR9, CCR10, CX3CR1, CXCR1 and CXCR4—as well as GPR4 and GPR182. These associations underscore the versatility of RAMP1 in modulating multiple signaling networks relevant to both physiological homeostasis and disease progression, including cancer [4].

Understanding the function of RAMPs is essential due to their broad impact on GPCRs modulation. Insights from structural biology, especially those involving membrane protein complexes, have major implications for drug discovery targeting GPCRs and for deciphering the pharmacology, biology and regulatory mechanisms of these receptors [4].

### Crosstalk With Tumor-Associated Receptors such as EGFR and VEGFR

Beyond its role as a GPCR accessory protein, accumulating evidence suggests that RAMP1 may participate in higher-order signaling integration, converging with receptor tyrosine kinase pathways central to tumor angiogenesis and tissue remodeling. Although most research focuses on RAMP associations with Class B GPCRs such as CALCRL, knockout models indicate that several phenotypes may arise from the coordinated activity of multiple GPCRs/RAMP complexes, broadening the potential for cross-talk with additional signaling pathways [4].

In RAMP1-deficient mice, wound-induced angiogenesis and lymphangiogenesis were impaired, accompanied by reduced expression of VEGF-A, VEGF-C and VEGFR-3, as well as defective lymphatic vessel formation and diminished interstitial drainage [12]. Similar findings were reported in models where RAMP1 deficiency led to persistent lymphedema, reduced lymphangiogenesis and lower expression of VEGF-C and VEGFR-3 distal to lymphatic injury sites, alongside decreased recruitment of pro-inflammatory macrophages [13]. These results reinforce the functional connection between RAMP1 and the VEGF/VEGFR axis and highlight its role in angiogenic and inflammatory processes linked to tumor growth and dissemination.

Additional studies support a broader relationship between RAMP1 and signaling pathways governing cell proliferation and tissue remodeling—processes that share components with receptor tyrosine kinases such as EGFR. For example, RAMP1 overexpression stimulates fibroblast proliferation and increases expression of G-protein signaling proteins and the Yes-associated protein (YAP), suggesting potential involvement in proliferative circuits analogous to those activated by EGFR [14]. Furthermore, the CALCRL/RAMP1 complex exhibits mechanosensitive properties in macrophages, where CGRP/RAMP1 signaling contributes to extracellular matrix homeostasis [4]. Taken together, both processes appear to be relevant to tumor microenvironment (TME) remodeling and suggest a multifaceted role of RAMP1 in integrating multiple signaling pathways [4].

Predictive protein–protein interaction analyses provide computational support for these hypotheses. STRING network modeling indicates that CALCRL/RAMP1 and receptors such as EGFR and VEGFR converge on shared potential signaling nodes, including MAPK/ERK, PI3K and transcriptional regulators such as YAP [15]. BioGRID analysis likewise identifies overlapping downstream components situating GPCRs/RAMP complexes within modules of proliferation, angiogenesis and tissue remodeling that are shared with receptor tyrosine kinases [16].

Together, these findings suggest that RAMP1 may serve as an integrative hub linking GPCRs and growth-factor receptor signaling, influencing angiogenesis, cell proliferation and local immune responses. This crosstalk suggests that GPCRs/RAMP complexes may exert substantial effects in tumor-associated processes including vascular growth, tissue remodeling and inflammation [4].

## Multi-omics Strategies Applied To Oncology

### Definitions of the Transcriptome, Proteome, Epigenome, Metabolome, and Interactome

The transcriptome refers to the complete set of RNA molecules expressed in a cell or tissue under a specific biological condition. It includes mRNAs, rRNAs, tRNAs, and non-coding RNAs, which play essential roles in fine-tuning gene expression and regulating post-transcriptional networks that respond to physiological and pathological stimuli [17]. Technologies such as RNA-seq expanded the field by enabling the identification of differential expression profiles and transcriptional signatures associated with tumor phenotypes. In the context of RAMP1, transcriptomics can reveal regulatory patterns linked to inflammatory microenvironments, hypoxia, or neuroendocrine stimuli, indicating whether its expression is associated with angiogenesis, immune evasion, or therapeutic resistance.

Complementing these findings, the proteome represents the full set of proteins within a biological system, including isoforms, structural variants, and post-translational modifications that determine protein stability, cellular localization, and function [18]. Unlike the transcriptome, the proteome reflects the functional execution of the cellular program and may diverge considerably from gene expression patterns. Proteomics approaches based on mass spectrometry allow investigation of RAMP1’s conformational structure, ligand affinity, and formation of stable complexes with GPCRs—particularly CALCRL—that modulate the CGRP pathway and influence tumor processes dependent on neuroimmune signaling.

Additionally, the epigenome comprises chemical modifications such as DNA methylation, histone modifications, and the three-dimensional organization of chromatin that regulate gene expression without altering the nucleotide sequence [19]. These marks constitute a dynamic and adaptive regulatory layer, sensitive to environmental stimuli and intracellular signaling. Integrating epigenomic analysis into RAMP1 research enables evaluation of whether promoter hypermethylation, histone acetylation, or enhancer reorganization modulate its expression in tumors. Such events may contribute to aggressive phenotypes, maintenance of inflammatory niches, or metabolic adaptation.

The metabolome corresponds to the complete set of detectable metabolites within a cell, tissue, or organism. It provides a functional snapshot of the biochemical state, shaped by the integration of genetic, proteomic, and epigenetic regulation [20]. In oncology, metabolomics helps identify alterations in pathways such as aerobic glycolysis, lipid metabolism, and the production of inflammatory mediators. Applied to RAMP1, this layer may reveal how activation of the CGRP/RAMP1 axis is linked to metabolic phenomena related to tumor growth, stromal remodeling, and interaction with the immune system.

Finally, the interactome encompasses the complete network of interactions among proteins, nucleic acids, and metabolites that sustain cellular processes across multiple scales [21]. Mass spectrometry–based methods, multiprotein complex purification, and computational modeling enable the identification of physical and functional interactions, revealing critical biological dependencies. For RAMP1, interactome mapping is essential to understand its integration into signaling circuits linked to neuroinflammation, angiogenesis, and cell–microenvironment communication—processes central to malignant progression and potential therapeutic sensitivity.

### Technologies for Generating and Integrating Omics Data: RNA-seq, MS, ChIP-seq, ATAC-seq, scRNA-seq, and Spatial Transcriptomics

Advances in sequencing and molecular analysis technologies have fundamentally transformed our understanding of biological systems. Methods such as RNA-seq, mass spectrometry (MS), ChIP-seq, ATAC-seq, scRNA-seq (single-cell RNA sequencing), and spatial transcriptomics allow integrated mapping of multiple levels of genetic, epigenetic, and proteomic regulation. These tools form the foundation of multi-omic studies, where distinct layers of biological information are combined to reveal complex molecular mechanisms that cannot be understood in isolation [22–24].

RNA-seq is the reference method for global quantification of gene expression. It relies on next-generation sequencing (NGS) of complementary DNA (cDNA) fragments obtained from total RNA, enabling the identification of expressed genes, transcript isoforms, alternative splicing events, and gene fusions [22]. Recent developments include total RNA-seq (capturing coding and non-coding RNAs), poly(A)-seq (targeting mature mRNAs), and Ribo-seq, which assesses only transcripts actively translated into proteins. Long-read sequencing technologies, such as PacBio Iso-Seq and Oxford Nanopore Direct RNA-seq, now allow full-length transcript mapping, improving the characterization of transcriptomic diversity [23].

ChIP-seq (Chromatin Immunoprecipitation Sequencing) identifies genomic regions bound by transcription factors and modified histones, revealing the functional landscape of chromatin [25]. ATAC-seq (Assay for Transposase-Accessible Chromatin) maps accessible DNA regions, highlighting active promoters and enhancers [26]. Integrating ChIP-seq and ATAC-seq provides insight into how chromatin structure directly influences gene expression.

ScRNA-seq enables the study of gene expression at single-cell resolution, revealing cellular heterogeneity within complex tissues [27]. It is essential for identifying distinct cell populations in tumors, organs, and developing systems, and for mapping differentiation trajectories and immune responses.

Spatial transcriptomics combines RNA sequencing with spatial localization of cells, allowing visualization of where each gene is expressed within a tissue [28, 29]. Methods such as Visium (10x Genomics), MERFISH, and Slide-seqV2 link molecular profiles with tissue architecture and are widely applied in oncology, neuroscience, and developmental biology. Integration with scRNA-seq produces a three-dimensional map of gene expression, correlating molecular signatures with cell morphology.

### Integration of Public Datasets: TCGA, GTEx, CPTAC and CCLE

Integration of standardized public datasets is a central pillar of contemporary multi-omic research. The Cancer Genome Atlas (TCGA) provides large-scale omics data, including RNA-seq, DNA methylation, genomic variants, and mass-spectrometry-based proteomics. For transcriptomic analyses, TCGA offers hundreds of tumor samples per subtype, often paired with adjacent normal tissue when available [30, 31].

To characterize gene expression in healthy tissues, researchers use the Genotype-Tissue Expression (GTEx) project, which includes thousands of normalized RNA-seq samples across more than 50 tissue types. Integration of TCGA and GTEx typically uses harmonized pipelines such as Toil/Recount2 to reduce cross-platform technical variation, as described in methodological studies [30, 31].

Preprocessing workflows involve filtering low-expression genes, performing quality control (QC), and removing outlier samples using unsupervised methods such as PCA or hierarchical clustering. Widely accepted approaches for normalization and differential expression include TMM (edgeR) and VST/DESeq2, which are correct for sequencing depth and statistical dispersion. Common analytical thresholds include:

- Statistical significance: FDR (Benjamini–Hochberg) < 0.05.
- Effect size: |log₂FC| ≥ 1 (or stricter, depending on dataset).

The Clinical Proteomic Tumor Analysis Consortium (CPTAC) provides quantitative proteomics matched to the same TCGA samples, enabling correlation between RNA levels, protein abundance, and post-translational modifications. The Cancer Cell Line Encyclopedia (CCLE) contributes multi-omic profiles of cancer cell lines, serving as experimental models for in vitro functional validation. Despite the robustness of these repositories, it is important to consider that bulk multi-omics data from TCGA represent a mixture of cell types, which may mask cell-specific signatures. Therefore, integrating single-cell and spatial technologies is a crucial step to validate these findings within the complex architecture of the tumor microenvironment.

Together, these repositories support a systematic and comparative approach to biomarker discovery, prediction modeling, and identification of new therapeutic targets in precision oncology [30].

## Differential Expression of RAMP1 in Various Types of Cancer

### Pan-Cancer Analysis of RAMP1 Expression (TCGA/GTEx)

Pan-cancer profiling provides a broad view of RAMP1 expression across distinct tumor types and highlights its context-dependent roles in cancer biology. This comparative landscape shows that RAMP1 behaves differently according to the affected tissue, underscoring the importance of using these datasets to identify shared and divergent molecular patterns. Such insights may support the identification of potential biomarkers and candidates for further evaluation as a therapeutic target [1].

### Comparisons Between Normal and Tumor Tissues

Data from TCGA and the GTEx project demonstrate that the expression of RAMP family members, including RAMP1, varies substantially between normal and tumor tissues [9]. In hepatocellular carcinoma (LIHC), RAMP1 is markedly upregulated, which may be associated with a microenvironment conducive to tumor progression. In contrast, renal cell carcinoma (KIRC) shows reduced RAMP1 expression [1], a pattern associated with increased infiltration of memory CD4^+^ T cells [9], raising the possibility of a context-specific suppressive role.

Clinical parameters such as tumor stage, prognosis and overall survival also correlate with altered RAMP1 expression. Higher levels have been associated with poorer outcomes [32], further highlighting RAMP1 as a potential prognostic biomarker or a candidate for therapeutic evaluation target. It is important to note that RAMP1 participates in physiological processes involving neurovascular regulation, inflammation, angiogenesis and neuroimmune communication, all of which influence the TME [33].

### Correlations With Molecular Subtypes and Clinical Stages

Given its heterogeneous biological roles, the impact of RAMP1 varies according to tumor subtype. In colorectal cancer, this complexity is evident. The gastrointestinal tract is densely innervated, and this neural network supports tumor spread. Tumor analyses show that RAMP1 expression is more strongly associated with microsatellite instability (MSI) tumors than with chromosomal instability (CIN) or genomically stable (GS) subtypes [34].

This association may be partially attributed to the intense immune infiltration characteristic of MSI tumors, since immune cells are major contributors to RAMP1 expression. Clinically, this corresponds to poorer overall survival in patients with MSI tumors and a higher prevalence in individuals under 50 years of age. In this context, denervation strategies—surgical or chemical—have been explored to slow tumors [34].

Regarding clinical stage, elevated RAMP1 levels have been associated with more advanced disease, including higher T classifications. However, this relationship is not universal and likely depends on tumor subtype and microenvironmental features [1]. This reinforces the inherent heterogeneity of RAMP1 and the need for more granular molecular characterization to define its clinical implications.

## RAMP1 in the Context of the Tumor Transcriptome

### Co-expressed Genes with RAMP1 and Gene Regulatory Networks

Tumor transcriptomics remains a central tool for identifying cancer biomarkers and their genetic signatures. By analyzing RNA transcripts, it is possible to map the expression patterns used by malignant cells to proliferate, invade tissues, and survive. These analyses also help predict tumor aggressiveness and potentially assist in the development of personalized therapeutic strategies [35].

Among the pathways frequently altered in malignant tumors are those mediated by GPCRs, which regulate tumor growth and metastasis [36]. The functional specificity of these receptors depends on RAMP proteins, including RAMP1, which associates with CALCRL to form CGRP and adrenomedullin receptors—key regulators of vasodilation, inflammation, and cell growth [9, 37].

Because gene expression operates as a network, understanding the role of RAMP1 requires examining its interactions with other genes. TCGA-based studies show that RAMP1 is consistently co-expressed with a specific gene set across multiple cancer types [9]. Gene Set Enrichment Analysis (GSEA) indicates a predominant association with epithelial–mesenchymal transition (EMT), while Gene Ontology (GO) analyses reveal enrichment in extracellular matrix organization and cell adhesion—processes essential for invasion and metastasis [8, 9].

**Fig. 2:**
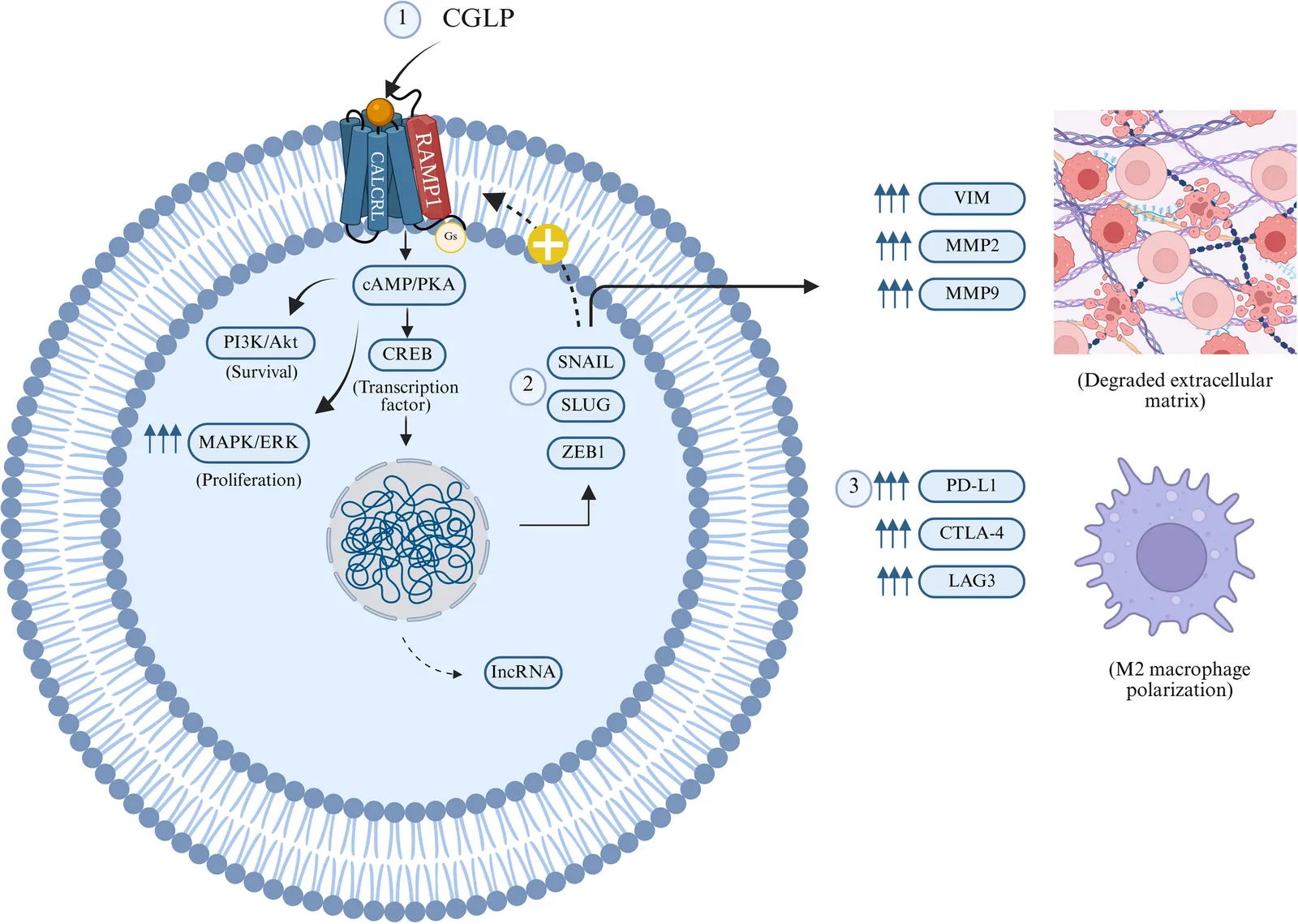
The RAMP1/CALCRL signaling axis is associated with tumor aggressiveness and immune microenvironment reprogramming. Binding of CGRP or Adrenomedullin (ADM) neuropeptides to the surface RAMP1/CALCRL receptor complex may trigger the activation of the coupled Gαs protein and adenylyl cyclase (AC), resulting in increased intracellular cAMP levels and subsequent activation of Protein Kinase A (PKA). PKA acts as a central node initiating divergent hierarchical signaling: [1] Activation of the PI3K/AKT and Ras/Raf/MEK/ERK (MAPK) pathways, promoting cell proliferation and survival [2]. Nuclear translocation and activation of CREB, potentially inducing Epithelial-Mesenchymal Transition (EMT) transcription factors, such as SNAIL and ZEB1, which upregulate Vimentin and metalloproteinases (MMPs), facilitating migration and invasion [3]. Establishment of an immunosuppressive microenvironment through the upregulation of immune checkpoints (PD-L1, CTLA-4, LAG3) and M2 macrophage polarization, potentially contributing to T cell exhaustion. Signaling is sustained by positive feedback loops involving transcription factors and lncRNAs that maintain RAMP1 expression

This co-expression network suggests the recruitment of pro-tumorigenic intracellular pathways downstream of the RAMP1/CALCRL complex, as is shown in Fig. 2. These include cAMP/PKA [7], PI3K/Akt, and MAPK [8], which promote proliferation, survival, and cell migration. As a result, malignant cells acquire increased proliferative autonomy, stress resistance, and enhanced capacity to remodel the extracellular matrix [9].

Although RAMP1 is often described as a structural modulator of CALCRL, functional evidence shows that its activation triggers a hierarchical signaling axis in which the cAMP/PKA pathway serves as the initial core. Binding of CGRP or adrenomedullin to the RAMP1/CALCRL complex rapidly increases cAMP levels and activates PKA [38, 39]. This early activation enables subsequent PI3K/Akt signaling and amplifies MAPK/ERK activity, forming a convergent axis that drives survival, proliferation, and tissue remodeling [40].

In the context of EMT, the cAMP/PKA axis induces CREB-dependent factors that upregulate mesenchymal regulators such as SNAIL and ZEB1 [41, 42]. At the same time, PI3K/Akt stabilizes these proteins, while MAPK enhances the transcriptional reprogramming associated with cell motility and invasion [42]. Similarly, RAMP1-driven immunosuppression relies on cooperation between these signaling pathways [43].

The cAMP/PKA pathway modulates the expression of CREB-dependent immunoregulatory molecules, including PD-L1 and LAG3. PI3K/Akt sustains the exhausted T-cell phenotype, and MAPK promotes low-grade inflammatory cytokines that facilitate the recruitment of M2 macrophages [44–46]. Together, these data suggest that cAMP/PKA may function as a primary signaling node that coordinates downstream Akt and MAPK signaling [47].

Beyond structural and motility-related pathways, RAMP1 shows strong correlations with tumor immune parameters. Its expression is associated with macrophage and T-cell infiltration, as well as upregulation of VEGFB, LAG3, and CTLA-4—markers of immune exhaustion [9], suggesting a role in establishing an immunosuppressive microenvironment.

### Inflammatory, Neurovascular, and Pro-Tumor Signatures

The EMT signature associated with RAMP1 features high expression of MMP2 and MMP9, which degrade the extracellular matrix, and Vimentin (VIM), a key marker of mesenchymal motility [8, 9, 48].

Angiogenesis is another critical component. The RAMP1-associated network links to a neurovascular signature involving adrenomedullin (ADM), a potent angiogenic factor, and VEGFB, which supports the stabilization of nascent vessels [9, 49].

**Fig. 3:**
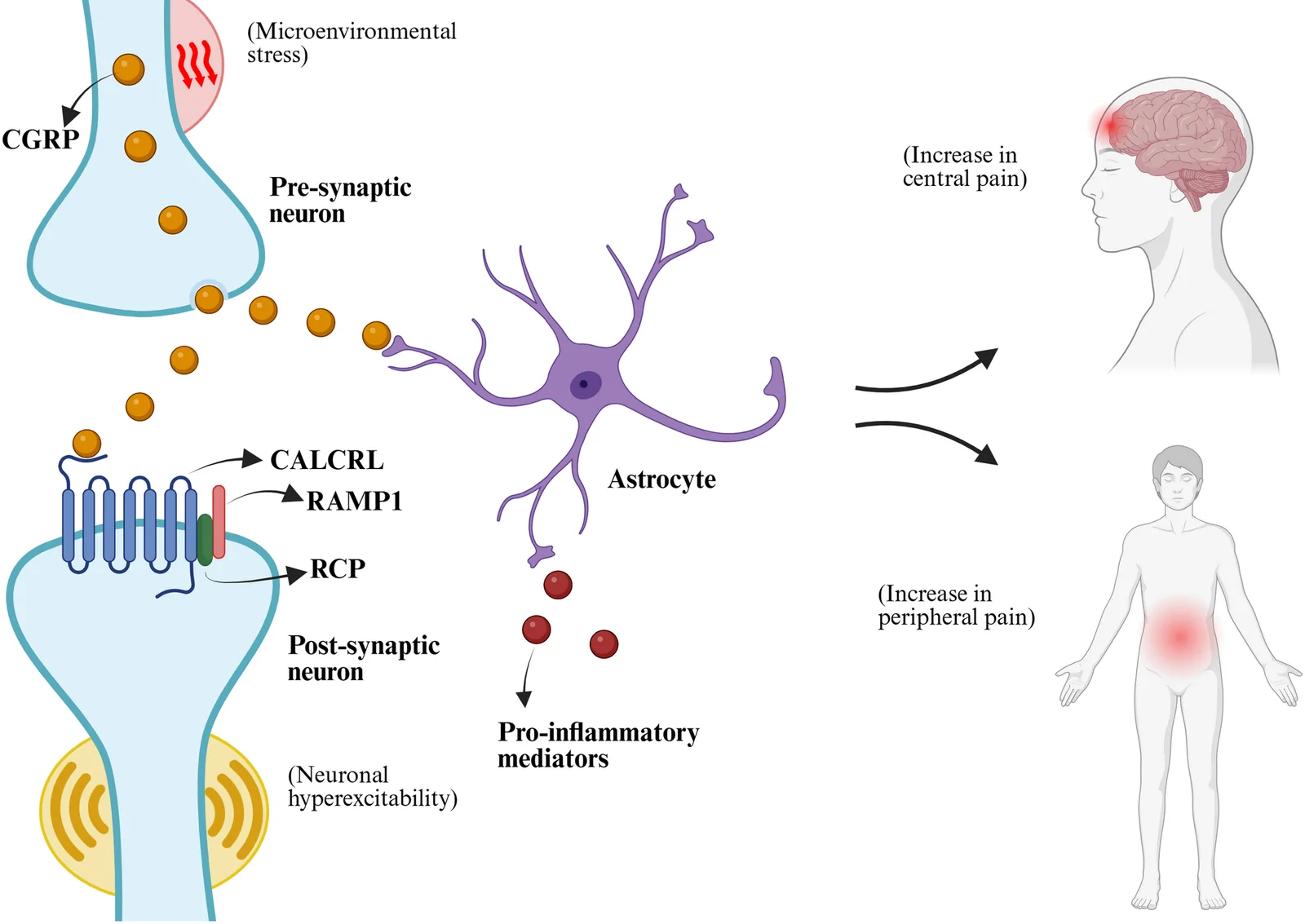
Schematic representation of CGRP in pain signaling. CGRP released from sensory nerve terminals binds to the CALCRL/RAMP1/RCP receptor complex on the postsynaptic neuron, contributing to nociceptive signal transmission. This signaling may promote neuronal hyperexcitability and stimulate astrocytes, which release pro-inflammatory mediators that contribute to hyperesthesia and hyperalgesia in the affected tissue

Within the inflammatory axis, the TME exhibits chronic low-grade inflammation. Cellular stress activates nerve terminals that release CGRP, which binds to the RAMP1/CALCRL complex and triggers neurogenic inflammation and increased vascularization, as illustrated in Fig. 3 [50]. This environment recruits M2 macrophages, which secrete pro-angiogenic cytokines [51]. This dynamic also supports increased expression of immune checkpoints such as CTLA-4 and LAG3, consolidating immune exhaustion [9, 51].

### Interactions with Transcription Factors and Non-Coding RNAs

Processes such as invasion, angiogenesis, and immune evasion rely on coordinated regulation by transcription factors (TFs). The strong association between RAMP1 and EMT suggests interactions with TFs such as SNAIL, SLUG, and ZEB1 [44]. RAMP1 signaling can activate these TFs, which also regulate RAMP1 expression, forming a positive feedback loop that reinforces malignancy [52, 53].

An additional regulatory layer involves non-coding RNAs. In several tumors, lncRNAs neutralize tumor-suppressive miRNAs through ceRNA mechanisms. In gastric cancer, the lncRNA LINC01133 sequesters miR-34a-5p, an inhibitor of EMT, thereby promoting tumor invasion [53]. This observation indicates that the pro-tumor activity of RAMP1 is supported through the integrated regulation of TFs and ncRNAs, facilitating the persistence of the malignant program.

## Epigenomics and Regulation of RAMP1 Expression

### DNA Methylation and Histone Modification Mechanisms Regulating RAMP1

The epigenomic landscape, which includes DNA methylation, modulates transcription and gene expression and plays a crucial role in disease processes [54]. RAMP1 is considered a key mediator linking hormonal fluctuations to CGRP signaling. Its expression can be regulated by estrogen-driven epigenetic mechanisms [55]. A significant correlation has been observed between RAMP1 expression and ESR2, the gene encoding the estrogen receptor ERβ. This finding suggests that ERβ signaling may influence the dynamics of the CGRP receptor [55].

DNA methylation typically occurs in promoter regions and is generally associated with chromatin compaction and gene silencing [55]. The promoter region of RAMP1 contains multiple CpG dinucleotides susceptible to methylation [55]. Evidence suggests that estrogen can influence RAMP1 expression through long-term DNA methylation effects [55]. Estrogen-induced hypermethylation of RAMP1 has been reported in transgender women undergoing hormonal therapy (17-β-estradiol) [55].

In addition, increased methylation at a specific CpG site within the RAMP1 promoter has been associated with disease physiopathology [55]. Conversely, lower methylation at a distinct CpG unit within the promoter region (positions + 89, +94, + 96 relative to the transcription start site, TSS) was significantly associated with a higher risk of a chronic neurological condition in females [55].

Histone modifications are essential for chromatin regulation [56]. Histone acetylation, such as H3K27ac, correlates with chromatin opening and transcriptional activation [56], and it is a defining mark of active enhancers [56]. Similarly, active histone methylation marks such as H3K4me3 are associated with transcriptional activation [56] and characterize promoter/TSS regions [56, 57]. In contrast, repressive marks such as H3K9me3 associate strongly with condensed heterochromatin [56], and H3K27me3, often found in regions depleted of H3K4me3, indicates transcriptional silencing [56, 57].

### ATAC-seq and ChIP-seq Data in Tumor Cell Lines

Although current sources do not report direct histone-modification data for the RAMP1 locus in tumor cell lines, the combined use of chromatin accessibility profiling (ATAC-seq) and histone-mark mapping (ChIP-seq) provides a powerful inferential framework to characterize regulatory landscapes and identify elements such as TSSs and enhancers [56, 57]. Epigenome sequencing using ChIP-seq and ATAC-seq enables dissection of the genome-wide regulatory landscape, revealing chromatin states and associated transcription factors [56].

ATAC-seq and ChIP-seq have been applied in studies of replicative senescence (RS) and oncogene-induced senescence (OIS) in human fibroblasts (2BS cells) [57]. This integrated multi-omic approach (ATAC-seq, RNA-seq, and ChIP-seq) identifies key regulatory elements, in which overlapping THSSs (ATAC-seq) and histone modifications (hPTMs) allow classification of regions as TSS-like (H3K4me3) or enhancer-like (H3K4me1 and H3K27ac) [57].

Regarding RAMP1 expression in tumor cell lines, bioinformatic analyses and qRT-PCR validation show that RAMP1 is elevated in OS cell lines, specifically MG63 and HOS, compared with the normal osteoblast line hFOB1.19 [9]. High RAMP1 expression has consistently been associated with poor prognosis across multiple cancer types (pan-cancer), including invasive breast carcinoma (BRCA), prostate adenocarcinoma (PRAD) and liver hepatocellular carcinoma (LIHC) [9].

### Effects of Epigenetic Modulators on RAMP1 Expression

Epigenetic modulation may link hormonal fluctuations to CGRP signaling, making RAMP1 epigenetic regulators a growing area of research [55]. In vitro and organ culture experiments using estradiol (E2) and the DNA methylation inhibitor 5-aza-2′-deoxycytidine (5-aza-dC) investigated the regulation of RAMP1 and CALCA (Calcitonin Gene-Related Peptide Alpha)—a gene that produces neuroendocrine peptides through alternative mRNA processing—in neuronal cells [55].

Estradiol stimulation increased RAMP1 expression and reduced CALCA expression in human neuronal cells (SK-N-DZ, a female-derived neuroblastoma cell line). Similar results occurred in organ cultures of trigeminal ganglia (TG) from male rats, where E2 increased RAMP1 and decreased CALCA expression [55].

Treatment with 5-aza-dC alone decreased RAMP1 expression and dramatically increased CALCA expression in human neuronal cells. In rat TG organ cultures, 5-aza-dC increased RAMP1 expression and showed a trend toward reduced CALCA expression (*p* = 0.08), though not significantly [55].

Combined estradiol and 5-aza-dC treatment rescued the RAMP1 response, resulting in increased RAMP1 expression. In contrast, CALCA expression increased more than 1000-fold in the human cell line. In rat TG, the combination upregulated both RAMP1 and CALCA [55].

This divergence between models (human cells vs. male rat TG) may reflect species-specific, sex-specific, or baseline methylation differences. However, the consistent observation that both RAMP1 and CALCA respond to combined E2 and 5-aza-dC treatment strongly suggests the presence of a methylation-sensitive regulatory mechanism [55]. The study reinforces the idea that sex hormones may induce RAMP1 hypermethylation, offering mechanistic insight into how estrogen modulates long-term gene expression [55].

## Single-Cell Data and Intratumoral Heterogeneity

The TME is a complex and dynamic entity composed of immune cells, stromal cells, blood vessels, and the extracellular matrix (ECM). Rather than acting as a passive bystander, the TME actively drives cancer progression [58]. The heterogeneity of gene expression and the plasticity of cells within the TME make single-cell–scale analyses indispensable for uncovering the specific roles of proteins such as RAMP1 [58].

### Expression of RAMP1 in Different Cellular Components of the Tumor Microenvironment (Malignant, Endothelial, Immune)

RAMP1 expression is observed in several cellular components of the TME, including malignant cells, endothelial cells, and immune cells. In malignant cells, RAMP1 is substantially expressed in OS lines such as MG63 and HOS when compared with the normal osteoblast line hFOB1.19 [9]. High RAMP1 expression in OS tumor cells has been linked to a metastatic phenotype and poor prognosis [9].

In immune cells, scRNA-seq analyses have revealed increased RAMP1 expression in TILs, particularly CD3⁺, CD4⁺, and FOXP3^−^ T cells in Head and Neck Squamous Cell Carcinoma (HNSCC) [59]. In human melanoma, scRNA-seq identified a population of CD8⁺RAMP1⁺ T cells that showed higher exhaustion levels compared with CD8⁺RAMP1⁻ cells [59]. Bulk analyses in OS using CIBERSORT estimated that high RAMP1 expression correlated positively with γδ T-cell infiltration and negatively with activated memory CD4⁺ T cells [9].

Regarding macrophages and neutrophils, RAMP1 expression in OS was significantly associated with markers of M2 macrophages (CD163 and VSIG4) and markers of neutrophils (ITGAM) [9]. For B cells and mast cells, RAMP1 expression correlated positively with activated mast cells (*r* > 0, *p* < 0.001) but negatively with memory B cells (*r* = − 0.26, *p* = 0.017) [9].

Endothelial cells (ECs) and cancer-associated fibroblasts (CAFs) are also essential stromal components [58]. Across pan-cancer datasets, high RAMP1 expression correlated positively with the Stromal Score in Colon Adenocarcinoma (COAD), Bladder Urothelial Carcinoma (BLCA), and Rectal Adenocarcinoma (READ) [9]. This score reflects stromal infiltration and serves as a biomarker of stromal contribution to tumor architecture [9]. In OS, however, the low-RAMP1 group showed a significantly higher Stromal Score than the high-RAMP1 group (*p* < 0.01) [9]. These differences highlight the heterogeneous stromal roles of RAMP1 across tumor types.

### scRNA-seq and Spatial Transcriptomics Analyses

High-resolution technologies are crucial because immune cells such as macrophages and neutrophils are shaped by factors like hypoxia and cytokine secretion within the TME, which can drive distinct phenotypes [58]. scRNA-seq has been used to characterize RAMP1 expression in immune subpopulations, including CD4⁺ and CD8⁺ T cells in HNSCC and melanoma [59]. These analyses indicated an increased RAMP1 expression in TILs and linked RAMP1 to CD8⁺ T-cell exhaustion [59].

Although the relative scarcity of integrated spatial transcriptomics data across diverse cancer types represents a notable limitation in the current characterization of RAMP1, its expression must be interpreted within a spatial context, as the localization of immune and malignant cells within the TME directly influences their function [58]. Spatial transcriptomics enables the correlation of RAMP1 expression with cellular organization and tissue architecture [58].

In the neuroimmune context, RAMP1-containing receptor complexes are present on sensory nerve fibers that infiltrate the TME [59]. Mapping the precise location of RAMP1⁺ nerve fibers and their direct interaction with immune and tumor cells would greatly benefit from spatial tools, as these interactions modulate antitumor immunity [59].

### Implications of Cellular Variability for RAMP1 Function

The heterogeneity of RAMP1 at the cellular level and its complex signaling imply multiple functional and prognostic roles. scRNA-seq showed that CD8⁺RAMP1⁺ T cells in melanoma display increased exhaustion [59]. However, survival studies suggested that patients with higher levels of CD8⁺RAMP1⁺ T cells had better outcomes [59]. This apparent contradiction reflects the complexity of RAMP1 signaling and the influence of tumor-specific contexts [59].

RAMP1 function is also linked to sensory perception and nociceptive signaling, which arise from tissue damage [59]. CGRP can promote immunosuppression and drive CD8⁺ T-cell exhaustion [59]. Pharmacologic RAMP1 inhibition has been shown to reduce T-cell exhaustion and suppress tumor growth, emphasizing that RAMP1 variability in neural and immune populations shapes local immunity and tumor progression [59].

CD8⁺RAMP1⁺ T cells may represent a subset that was initially strongly activated in response to the tumor but later became exhausted due to neuroimmune suppression and immune checkpoints within the TME [59]. The TME is a continuously evolving system that drives molecular, cellular, and physical changes [58]. Nociceptive signaling through CGRP/RAMP1 induces CD8⁺ T-cell exhaustion and inhibits effector function, promoting tumor progression and immune evasion [59]. In melanoma, RAMP1 blockade in mouse models reduced T-cell exhaustion and improved survival, supporting the proposed suppressive nature of the CGRP/RAMP1 axis [59].

It is possible that CD8⁺RAMP1⁺ T-cell exhaustion in melanoma is less profound or that these cells retain some latent functionality or long-term memory capacity. This may explain their association with improved outcomes, in contrast to other tumor types where RAMP1 promotes more direct tumor-supportive effects [59]. The presence of exhausted CD8⁺RAMP1⁺ T cells also indicates that antitumor immune surveillance occurred and persisted despite suppressive conditions [59].

In the specific context of melanoma, it is plausible to hypothesize that CD8⁺RAMP1⁺ T cells may function as markers of “past immune engagement”: they display exhaustion driven by the suppressive TME but also reflect strong previous infiltration, a feature associated with better survival [59].

Finally, high RAMP1 expression is consistently associated with poor prognosis in OS [9]. However, its correlation with the Stromal Score varies across cancers—positive in colon and rectal adenocarcinomas and bladder carcinoma, but inverse in OS, where low RAMP1 expression aligns with a higher Stromal Score [9]. This variability suggests that RAMP1 does not interact uniformly with stromal elements and that its pro- or anti-tumorigenic effects may depend on stromal origin or specific cell types such as ECs versus CAFs [58].

## RAMP1 and Treatment Response

As discussed previously, studies have demonstrated the presence of nerves innervating the TME [60], forming an intrinsic communication axis between tumors and immune cells [61]. This neuroimmune interface regulates essential processes, including tumor cell proliferation and expansion [60]. Recent evidence shows that nociceptor neurons within the TME release CGRP, which activates the CGRP/RAMP1 axis and promotes tumor growth, immune evasion, and microenvironmental remodeling [62].

These effects arise from nociceptor activity, which induces the secretion of neurotransmitters such as CGRP and substance P (SP). CGRP exerts its actions through a heterodimeric receptor composed of RAMP1 and CALCRL, activating the Gαs/adenylyl cyclase/cAMP signaling cascade. This pathway drives responses such as hematopoietic cell mobilization and the activation of pro-tumor signaling mechanisms [63]. In addition, recent studies demonstrate that the CGRP/RAMP1 axis modulates adaptive and inflammatory immune responses in peripheral tissues, reinforcing its regulatory role in immunity [64].

These factors are not only expressed within the TME but have also been correlated, in recent studies, with predictive biomarkers for the prognosis of patients with multiple cancer types [61, 65]. They are also directly involved in mechanisms of resistance to immunotherapy and chemotherapeutic agents [63]. Recent experimental findings suggest that RAMP1 signaling may impair NK-cell cytotoxicity and promote immune escape, contributing to therapeutic resistance [66]. Because of this critical influence on treatment response, RAMP1 has been proposed as a potential therapeutic target and a candidate predictive biomarker in oncology, highlighting the need for deeper investigation of its biological functions.

### RAMP1 as a Driver of Therapeutic Resistance

In cancer immunotherapy, immune checkpoint inhibitors (ICIs) have shown strong efficacy by blocking ligand–receptor interactions involving key inhibitory checkpoints on cytotoxic T cells, such as PD-1, LAG3, and TIM3 [67]. These receptors normally help maintain immune homeostasis by suppressing T-cell activity to prevent excessive responses. Tumor cells exploit these checkpoints through ligand expression, thereby sustaining immune evasion [65].

A correlation between low TME scores and improved responsiveness to ICI therapy has been demonstrated in genetically complex cancers such as OS and melanoma [61]. Within this context, the CGRP/RAMP1 axis was found to drive CD8⁺ T-cell exhaustion. Recent studies show that pharmacologic or genetic inhibition of this axis restores CD8⁺ T-cell cytotoxicity and enhances ICI responses, underscoring its relevance as a modulator of immunotherapy resistance [62].

Genetic ablation or pharmacologic silencing of nociceptor neurons, as well as blockade of the CGRP/RAMP1 axis, has been associated with reduced expansion of B16F10 tumors and preservation of antitumor immunity by preventing CD8⁺ T-cell exhaustion. These findings strengthen the view that CGRP/RAMP1 is a key therapeutic target capable of improving ICI responsiveness [65].

Another therapeutic strategy in oncology is stem-cell–based treatment, which involves the mobilization of hematopoietic stem cells (HSCs) to enable collection, transplantation, and subsequent hematopoietic and immune reconstitution [68]. The CGRP/RAMP1 axis plays a critical role in this process by mobilizing HSCs through cAMP-mediated signaling. It promotes HSC release without altering bone-marrow cellularity, acting functionally analogous to granulocyte colony-stimulating factor (G-CSF). In murine models, RAMP1 deletion selectively impaired HSC mobilization to peripheral blood. RAMP1 involvement was further demonstrated in pharmacologic studies: during plerixafor administration—an HSC-mobilizing agent—CGRP exhibited enhanced synergy when combined with G-CSF, improving stem-cell yield for autologous transplantation. In the context of chemotherapy, CGRP administration reversed cisplatin-induced deficits in hematopoietic stem and progenitor cell (HSPC) mobilization [63]. These findings reinforce RAMP1 as a strong candidate for combinatorial therapeutic strategies in cancer.

### RAMP1 as a Prognostic and Predictive Biomarker

Understanding RAMP1 as a TME component with immunosuppressive activity has prompted investigations into its prognostic value. The Tumor Microenvironment-Based Prognostic Index (TMEindex), which includes RAMP1 as part of its signature, was identified as an unfavorable prognostic biomarker across nine tumor types within OS, correlating with lower disease-free survival and increased five-year metastatic risk [61]. Additionally, high RAMP1 expression has been associated with poorer prognosis in OS and adverse clinical outcomes in READ, where it correlates with shorter disease-free and metastasis-free survival [9]. Pan-cancer analyses further highlight that elevated RAMP1 levels correlate with aberrant methylation patterns, infiltration of immunosuppressive cells, and worse overall survival across multiple solid tumors [69].

In prostate cancer, RAMP1 expression is particularly relevant because it is a direct target of NKX3.1, a prostate-specific transcription factor. Loss of NKX3.1 is present in approximately 20% of human prostatic intraepithelial neoplasia lesions and 40% of prostate tumors. Under these conditions, RAMP1 deletion markedly reduced proliferation and tumorigenicity. These findings support a role for NKX3.1 in suppressing RAMP1 expression and prostate tumorigenesis, highlighting its potential as a prognostic biomarker [8].

In aggressive tumors such as Ewing sarcoma (EwS)—a malignant bone and soft-tissue tumor with poor prognosis—the suppression of CALCB or RAMP1 produced antiproliferative effects in EwS cells both in vitro and in vivo. Notably, RAMP1 suppression had a stronger inhibitory impact on tumorigenicity, underscoring its value as a potential therapeutic target [70].

## Integration of Multi-Omic Data: Challenges and Tools

### Multi-Omics Integration Tools in Precision Medicine

Technological advances in healthcare have expanded the use of deep molecular analyses, including multi-omics datasets, which are essential in oncology due to the genetic and phenotypic complexity of cancer. This complexity creates challenges in understanding tumor development, predicting treatment response and estimating recurrence risk [71]. Multi-omics approaches help address these gaps by providing detailed insights into mutational processes, gene expression programs and metabolic alterations unique to each patient’s tumor, potentially enabling the identification of clinically relevant subgroups and supporting the development of more personalized treatment strategies [72].

In this context, several statistical frameworks have been developed to operationalize precision medicine. Among them, Multi-Omics Factor Analysis (MOFA) has emerged as a prominent unsupervised method for integrating multiple omics layers [73]. In a recent computational study of high-risk neuroblastoma, MOFA identified six genes associated with increased malignancy potential, including RAMP1. The model stratified tumor samples into two groups: an ultra-high-risk (UHR) cluster with poor survival and a common high-risk (CHR) cluster with significantly better outcomes. This illustrates the potential of MOFA in generating prognostic frameworks that may guide therapeutic decision-making [74].

Similar studies have identified RAMP1 as a potential prognostic factor in gastric cancer [32, 75] and OS [76]. Despite these advances in understanding GPCRs signaling, including RAMP1-mediated pathways, the field still faces important gaps. Consequently, artificial intelligence and deep learning tools are increasingly being applied to overcome longstanding challenges in cancer biology and to accelerate the discovery of novel prognostic and therapeutic targets [77].

### Current Challenges and Points of Conflict in the Application of Multi-Omics Approaches to Cancer Research

Although multi-omics technologies have transformed cancer research by expanding the understanding of cellular pathways, several limitations remain. These include the scarcity of large and diverse datasets, heterogeneity across available data sources and the need for extensive preprocessing, which may lead to loss of information [78].

Another major limitation is the predominance of purely in silico studies, which delays practical validation of computational findings [32, 79]. A recurring issue is the “curse of dimensionality,” where the number of variables vastly exceeds the number of samples, complicating the identification of true biological signals and favoring the accumulation of noise [60].

Likewise, in vitro analyses provide important functional clues but do not always reproduce in vivo behavior. For example, in a study evaluating RAMP1 and CALCB knockdown in Ewing sarcoma models, the antiproliferative effects were much stronger in vitro than in vivo [70]. Although both models supported the involvement of the gene pair, the incomplete in vivo response suggested insufficient knockdown or additional compensatory mechanisms, highlighting intrinsic limitations of animal models as well.

Epigenomic and spatial transcriptomic approaches may help bridge some of these gaps. The loss of the transcriptional repressor Prdm12, for instance, reduces RAMP1 expression and reverses T-cell exhaustion, linking RAMP1 to regulatory immune circuits [80]. In oral squamous cell carcinoma, scRNA-seq revealed enriched expression of RAMP1 and CALCRL among fibroblasts and immune cells [33]. However, the absence of spatial transcriptomic analyses limited the interpretation of these findings, emphasizing the need for future studies that incorporate spatial resolution to elucidate the architecture of the TME.

## Outlook - Challenges for Developing RAMP1-Targeted Therapies

Despite recent advances highlighting the CGRP/RAMP1 axis in tumor progression, immune modulation, and its emergence as a therapeutic target across different oncological contexts, the translation of these findings into clinical interventions remains at an early stage and faces substantial challenges.

One of the major obstacles is pharmacological selectivity, given the broad functional heterogeneity of RAMP1 in modulating multiple GPCRs. Systemic activation or inhibition of these receptors could potentially increase the risk of off-target effects with clinically relevant consequences [81]. Toxicity is another critical concern, as RAMP1 blockade interferes with essential physiological processes—including vascular regulation, nociception, and immune homeostasis—which may compromise therapeutic safety [82]. The blood–brain barrier (BBB) further complicates drug development, since a significant portion of CGRP/RAMP1 signaling occurs within central neurogenic pathways, requiring therapeutic strategies that enable selective permeation or precise molecular targeting [83].

Taken together, these factors indicate that, although RAMP1 is a highly promising pharmacological target, its translational advancement depends on the development of more selective pharmacological approaches, robust preclinical validation, and structural optimization to achieve greater target specificity and long-term safety in experimental models. Progress in this field is expected to arise from the convergence of pharmacology, biotechnology, and advanced multi-omic technologies, combined with functional in vivo validation and the design of therapeutic strategies that balance antitumor efficacy with systemic safety.

## Conclusion

### The Multifaceted Role of RAMP1 Revealed Through Multi-Omic Approaches

The integration of multi-omic data has advanced the understanding of RAMP1 beyond its classification as a mere receptor-associated component, positioning it as a versatile allosteric modulator of GPCRs. This expanded perspective highlights its involvement in promoting the EMT, activating key pro-tumorigenic pathways (PI3K/Akt, MAPK, and cAMP/PKA), and promoting phenotypes linked to invasion, metastasis, and microenvironmental remodeling.

Pan-cancer analyses further confirm the marked heterogeneity of RAMP1 expression across tumor types, with contexts of both overexpression—such as in hepatocellular carcinoma—and gene downregulation, as observed in renal cell carcinoma. This functional plasticity reinforces the need for tissue-specific analyses and precision oncology strategies to contextualize RAMP1’s role across diverse biological settings.

### Knowledge Gaps and Priorities for Functional Validation

Despite considerable progress, key challenges remain. The functional heterogeneity of RAMP1 requires more refined experimental models capable of capturing its spatial and intercellular dynamics. In this regard, the application of spatial transcriptomics is a priority, as it enables the integration of gene expression with tissue architecture and cellular niches within the TME—factors essential for understanding RAMP1 activity in vivo.

Uncertainties also persist regarding the epigenetic mechanisms regulating RAMP1 expression, particularly its interactions with canonical EMT transcription factors (SNAIL, SLUG, and ZEB1) and the post-transcriptional regulation mediated by non-coding RNAs. Detailed elucidation of these processes is fundamental to understanding how invasive tumor programs are activated, directed, and maintained.

### Therapeutic and Biomarker Potential of RAMP1 in Personalized Oncology

The association between high RAMP1 expression, CD8⁺ T-cell exhaustion, and reduced responsiveness to ICIs underscores its potential value as a predictive biomarker in immunotherapy. Conversely, its role in hematopoietic stem cell mobilization demonstrates therapeutic relevance in chemotherapeutic and transplant settings.

The established clinical use of CGRP inhibitors for migraine treatment expands the prospect of their optimization and pharmacological repurposing in oncology, particularly in tumors where RAMP1 functions as a poor prognostic biomarker, such as osteosarcoma, Ewing sarcoma, rectal adenocarcinoma, prostate cancer, and urothelial carcinoma. Nonetheless, such translational efforts must progress in alignment with the broader challenges of drug development—including selectivity, toxicity, and blood–brain barrier permeability—as discussed in the Outlook section.

### Final Considerations

By integrating multiple layers of multi-omic evidence, this review highlights RAMP1 as a significant axis in tumor progression and immune modulation, while establishing conceptual and mechanistic foundations to guide the development of innovative therapeutic strategies. Advances in this field will rely on the convergence of in vivo functional validation, spatially resolved technologies, and refined pharmacological engineering, ultimately paving the way toward more precise and effective interventions in contemporary oncology.

## Data Availability

No datasets were generated or analysed during the current study.

## References

[1] Yang,S., Huan, R., Deng, M., Luo, T., Peng, S., & Xiong, Y. (2024). Pan-cancer analysis revealed prognosis value and immunological relevance of ramps. *Heliyon* 10(3). e24849. 10.1016/j.heliyon.2024.e24849 38317990 PMC10838762

[2] Demir Karaman, E., & Işık, Z. (2023). Multi-omics data analysis identifies prognostic biomarkers across cancers. *Med Sci (Basel)*. 11(3):44. 10.3390/medsci11030044 37489460 PMC10366886

[3] Pioszak, A. A., & Hay, D. L. (2020). RAMPs as allosteric modulators of the calcitonin and calcitonin-like class B G protein-coupled receptors. In: Langmead CJ, editor. From Structure to Clinical Development: Allosteric Modulation of G Protein-Coupled Receptors,*Advances in Pharmacology . Vol. 88* . Academic Press; 2020. p. 115-141. 10.1016/bs.apha.2020.01.001 PMC7374980 32416865

[4] Kotliar, I. B., Lorenzen, E., Schwenk, J. M., Hay, D. L., & Sakmar, T. P. (2023). Elucidating the interactome of G protein-coupled receptors and receptor activity-modifying proteins. *Pharmacological Reviews*, *75*(1), 1–34. 36757898 10.1124/pharmrev.120.000180 PMC9832379

[5] Sahani, P. A., Patra, R., & Dixit, A. (2025). G protein-coupled receptors in cancer prognosis and therapeutics: A comprehensive review. *International Journal of Biological Macromolecules*, *328*, 147665. 40953624 10.1016/j.ijbiomac.2025.147665

[6] Zhou, W., Xia, H., Zeng, T., Tan, H., Zhang, Y., Li, Y., et al. (2025). Mechanisms and opportunities of the arrestin beta signaling pathway in liver diseases. *Dna and Cell Biology*, *44*(12), 647–661. 41125398 10.1177/10445498251381186

[7] Shao, L., Chen, Y., Zhang, S., Zhang, Z., Cao, Y., Yang, D., et al. (2022). Modulating effects of ramps on signaling profiles of the glucagon receptor family. *Acta Pharm Sin B*, *12*(2), 637–650. 35256936 10.1016/j.apsb.2021.07.028 PMC8897147

[8] Logan, M., Anderson, P. D., Saab, S. T., Hameed, O., & Abdulkadir, S. A. (2013). RAMP1 is a direct NKX3.1 target gene up-regulated in prostate cancer that promotes tumorigenesis. *American Journal of Pathology*, *183*(3), 951–963. 23867798 10.1016/j.ajpath.2013.05.021 PMC3763771

[9] Xie,L., Xiao, W., Fang, H., & Liu, G. (2023). RAMP1 as a novel prognostic biomarker in pan-cancer and osteosarcoma. *PLoS One*. 18(10), e0292452. 10.1371/journal.pone.0292452 37796823 PMC10553254

[10] Kliuchnikova,A., Gordeeva, A., Abdurakhimov, A., Materova, T., Tarbeeva, S., & Sarygina, E. (2025). Ovarian cancer: multi-omics data integration. *International Journal Of Molecular Sciences*, *26*(13), 5961. 10.3390/ijms26135961 40649740 PMC12249510

[11] Gluexam, T., Grandits, A. M., Schlerka, A., Nguyen, C. H., Etzler, J., Finkes, T., et al. (2019). CGRP signaling via CALCRL increases chemotherapy resistance and stem cell properties in acute myeloid leukemia. *International Journal of Molecular Sciences*, *20*(23), 5826. 31756985 10.3390/ijms20235826 PMC6928760

[12] Kurashige, C., Hosono, K., Matsuda, H., Tsujikawa, K., Okamoto, H., & Majima, M. (2014). Roles of receptor activity-modifying protein 1 in angiogenesis and lymphangiogenesis during skin wound healing in mice. *The Faseb Journal*, *28*(3), 1237–1247. 24308973 10.1096/fj.13-238998

[13] Mishima, T., Ito, Y., Nishizawa, N., Amano, H., Tsujikawa, K., Miyaji, K., et al. (2017). RAMP1 signaling improves lymphedema and promotes lymphangiogenesis in mice. *Journal of Surgical Research*, *219*, 50–60. 29078910 10.1016/j.jss.2017.05.124

[14] Yin,S., Song, R., Ma, J., Liu, C., Wu, Z., & Cao, G. (2022). Receptor activity-modifying protein 1 regulates mouse skin fibroblast proliferation via the Gαi3–PKA–CREB–YAP axis. *Cell Commun Signal*, *20*(1), 116. 10.1186/s12964-022-00852-0 35413847 PMC9004193

[15] Szklarczyk, D., Gable, A. L., Lyon, D., Junge, A., Wyder, S., Huerta-Cepas, J., et al. (2019). STRING v11: protein–protein association networks with increased coverage. *Nucleic Acids Research*, *47*(D1), D607–D613. 30476243 10.1093/nar/gky1131 PMC6323986

[16] Roychowdhury, T., & Abyzov, A. (2019). Chromatin organization modulates the origin of heritable structural variations in the human genome. *Nucleic Acids Research*, *47*(6), 2766–2777. 30773596 10.1093/nar/gkz103 PMC6451188

[17] Monzó, C., Liu, T., & Conesa, A. (2025). Transcriptomics in the era of long-read sequencing. *Nature Reviews Genetics*, *26*, 681–701. 40155769 10.1038/s41576-025-00828-z

[18] Coorssen,J. R., & Padula, M. P. (2024). Proteomics: The state of The field. *Proteomes*, *12*(2), 14. 10.3390/proteomes12020014 38651373 PMC11036260

[19] Bell,C. G. (2024). Epigenomic insights into common human disease pathology. *Cellular And Molecular Life Sciences*, *81*, 5206. 10.1007/s00018-024-05206-02 PMC11008083 38602535

[20] Moco, S., & Buescher, J. M. (2022). Metabolomics: going deeper, going broader, going further. In: *Methods in Molecular Biology*. pp. 155–178. 10.1007/978-1-0716-2624-5_11 36178626

[21] Wu, S., Zhang, S., Liu, C. M., Fernie, A. R., & Yan, S. (2025). Recent advances in mass spectrometry-based protein interactome studies. *Molecular and Cellular Proteomics*, *24*(1), 100887. 39608603 10.1016/j.mcpro.2024.100887 PMC11745815

[22] Gondane, A., & Itkonen, H. M. (2023). Revealing the history and mystery of RNA-seq. *Current Issues in Molecular Biology*, *45*(3), 1860–1874. 36975490 10.3390/cimb45030120 PMC10047236

[23] Ament, I. H., DeBruyne, N., Wang, F., & Lin, L. (2025). Long-read RNA sequencing: A transformative technology. *Molecular Therapy*, *33*(3), 883–894. 39563027 10.1016/j.ymthe.2024.11.025 PMC11897757

[24] Iadarola, P. (2022). Mass spectrometric proteomics 2022. *International Journal of Molecular Sciences*, *23*(22), 14246. 36430719 10.3390/ijms232214246 PMC9698491

[25] Nakato, R., & Sakata, T. (2021). Methods for ChIP-seq analysis. *Methods*, *187*, 44–53. 32240773 10.1016/j.ymeth.2020.03.005

[26] Grandi, F. C., Modi, H., Kampman, L., & Corces, M. R. (2022). Chromatin accessibility profiling by ATAC-seq. *Nature Protocols*, *17*, 1518–1552. 35478247 10.1038/s41596-022-00692-9 PMC9189070

[27] Jovic,D., Liang, X., Zeng, H., Lin, L., Xu, F., & Luo, Y. (2022). Single-cell RNA sequencing technologies and applications. *Clin Transl Med*. 12(3), e694. 10.1002/ctm2.694 35352511 PMC8964935

[28] Williams, C. G., Lee, H. J., Asatsuma, T., Vento-Tormo, R., & Haque, A. (2022). An introduction to Spatial transcriptomics. *Genome Medicine*, *14*(1), 68. 35761361 10.1186/s13073-022-01075-1 PMC9238181

[29] Jain, S., & Eadon, M. T. (2024). Spatial transcriptomics in health and disease. *Nature Reviews Nephrology*, *20*, 659–671. 38719971 10.1038/s41581-024-00841-1 PMC11392631

[30] Aguet, F., Anand, S., Ardlie, K. G., Gabriel, S., Getz, G. A., Graubert, A., et al. (2020). The GTEx consortium atlas of genetic regulatory effects across human tissues. *Science*, *369*(6509), 1318–1330. 32913098 10.1126/science.aaz1776 PMC7737656

[31] Jin, X., Mei, Y., Yang, P., Huang, R., Zhang, H., Wu, Y., et al. (2024). Prioritization of therapeutic targets using integrative multi-omics analysis. *Human Genomics*, *18*(1), 42. 38659038 10.1186/s40246-024-00571-2 PMC11040978

[32] Zhou,K., & Chen, R. (2025). Paraptosis-related classification and risk signature in gastric cancer. *Discover Oncol*, *16*(1), 2996. 10.1007/s12672-025-02996-0 PMC12170466 40524099

[33] Tu,N. H., Inoue, K., Lewis, P. K., Khan, A., Hwang, J. H., & Chokshi, V. (2023). Calcitonin-related polypeptide alpha mediates oral cancer pain. *Cells*, *12*(13), 1675. 10.3390/cells12131675 37443709 PMC10341289

[34] Parathan,P., Tran, K., Neil, L., Tan, T., Carli, A. L. E., & Liao, Y. (2025). Sensory neuropeptide CGRP and RAMP1 drive tumour cell growth. *BMJ Oncol*, 4(1), e000842. 10.1136/bmjonc-2025-000842 41158750 PMC12557745

[35] Supplitt,S., Karpinski, P., Sasiadek, M., & Laczmanska, I. (2021). Applications of transcriptomics in personalized cancer medicine. *Int J Mol Sci*. 22 :1422. 10.3390/ijms22031422 PMC7866970 33572595

[36] Chaudhary, P. K., & Kim, S. (2021). GPCRs and G-proteins as cancer drivers. *Cells*. 10 :3288. 10.3390/cells10123288 PMC8699078 34943797

[37] Hay, D. L., & Pioszak, A. A. (2016). Receptor activity-modifying proteins: New insights and roles. *Annual Review of Pharmacology and Toxicology*, *56*, 469–487. 26514202 10.1146/annurev-pharmtox-010715-103120 PMC5559101

[38] Russell, F. A., King, R., Smillie, S. J., Kodji, X., & Brain, S. D. (2014). Calcitonin gene-related peptide: Physiology and pathophysiology. *Physiological Reviews*, *94*(4), 1099–1142. 25287861 10.1152/physrev.00034.2013 PMC4187032

[39] Pawlak, J. B., Wetzel-Strong, S. E., Dunn, M. K., & Caron, K. M. (2017). Cardiovascular effects of adrenomedullin and CGRP. *Peptides*, *88*, 1–7. 27940069 10.1016/j.peptides.2016.12.002 PMC5706544

[40] Glaviano, A., Foo, A. S. C., Lam, H. Y., Yap, K. C. H., Jacot, W., Jones, R. H. (2023). PI3K/AKT/mTOR signaling pathway in cancer. *Mol Cancer*. 22 :1827. 10.1186/s12943-023-01827-6 PMC10436543 37596643

[41] Nieto, M. A., Huang, R. Y. J., Jackson, R. A., & Thiery, J. P. (2016). EMT: 2016. *Cell*, *166*(1), 21–45. 27368099 10.1016/j.cell.2016.06.028

[42] Zhang, H., Kong, Q., Wang, J., Jiang, Y., & Hua, H. (2020). Roles of cAMP–PKA–CREB signaling in cancer. *Exp Hematol Oncol*, *9*(1), 32. 33292604 10.1186/s40164-020-00191-1 PMC7684908

[43] Lamouille, S., Xu, J., & Derynck, R. (2014). Molecular mechanisms of epithelial–mesenchymal transition. *Nature Reviews Molecular Cell Biology*, *15*(3), 178–196. 24556840 10.1038/nrm3758 PMC4240281

[44] Dent, P., Curiel, D. T., Fisher, P. B., & Grant, S. (2009). Synergistic combinations of signaling pathway inhibitors. *Drug Resistance Updates : Reviews and Commentaries in Antimicrobial and Anticancer Chemotherapy*, *12*(3), 65–73. 19395305 10.1016/j.drup.2009.03.001 PMC2696566

[45] Wu, M., Huang, Q., Xie, Y., Wu, X., Ma, H., Zhang, Y., et al. (2022). Improvement of anticancer efficacy of PD-1/PD-L1 Blockade. *Journal of Hematology & Oncology*, *15*(1), 24. 35279217 10.1186/s13045-022-01242-2 PMC8917703

[46] Tsuru, S., Ito, Y., Matsuda, H., Hosono, K., Inoue, T., Nakamoto, S., et al. (2020). RAMP1 signaling in immune cells regulates inflammation-associated lymphangiogenesis. *Laboratory Investigation*, *100*(5), 738–750. 31911634 10.1038/s41374-019-0364-0

[47] Sasi, B., Ethiraj, P., Myers, J., Lin, A. P., Jiang, S., Qiu, Z., et al. (2021). Regulation of PD-L1 expression via cAMP-mediated immunosuppression. *Leukemia*, *35*(7), 1990–2001. 33299141 10.1038/s41375-020-01105-0 PMC8187478

[48] Ahmed, M. B., Alghamdi, A. A. A., Islam, S. U., Lee, J. S., & Lee, Y. S. (2022). cAMP signaling in cancer. *Cells*, *11*(13), 2020. 35805104 10.3390/cells11132020 PMC9266045

[49] Yadav, L., Puri, N., Rastogi, V., Satpute, P., & Sharma, V. (2015). Tumour angiogenesis and angiogenic inhibitors. *J Clin Diagn Res*, *9*, XE01–XE05. 26266204 10.7860/JCDR/2015/12016.6135 PMC4525594

[50] Lugano, R., Ramachandran, M., & Dimberg, A. (2020). Tumor angiogenesis. *Cellular and Molecular Life Sciences*, *77*, 1745–1770. 31690961 10.1007/s00018-019-03351-7 PMC7190605

[51] Zhang,Y., Lin, C., Wang, X., & Ji, T. (2020). Calcitonin gene-related peptide in oral squamous cell carcinoma. *Oncol Lett*, *20*, 12116. 10.3892/ol.2020.12116 PMC7509602 32994816

[52] Zhao,H., Wu, L., Yan, G., Chen, Y., Zhou, M., & Wu, Y. (2021). Inflammation and tumor progression. *Signal Transduct Target Ther*, *6*, 658. 10.1038/s41392-021-00658-5 PMC8273155 34248142

[53] Brabletz, S., & Brabletz, T. (2010). The ZEB/miR-200 feedback loop. *Embo Reports*, *11*, 670–677. 20706219 10.1038/embor.2010.117 PMC2933868

[54] Wan, D., Hou, L., Zhang, X., Han, X., Chen, M., Tang, W., et al. (2015). DNA methylation of RAMP1 gene in migraine. *The Journal of Headache and Pain*, *16*(1), 90. 26501962 10.1186/s10194-015-0576-7 PMC4623078

[55] Holm,A., Edvinsson, J. C. A., Krause, D. N., & Edvinsson, L. (2025). RAMP1-dependent regulation of CGRP. *The Journal Of Headache And Pain*, *26*(1), 2071. 10.1186/s10194-025-02071-7 PMC12172354 40528180

[56] Ma, S., & Zhang, Y. (2020). Profiling chromatin regulatory landscapes. *Mol Biomed*, *1*(1), 9. 34765994 10.1186/s43556-020-00009-w PMC7546943

[57] Song, Q., Hou, Y., Zhang, Y., Liu, J., Wang, Y., Fu, J., et al. (2022). Integrated multi-omics approach revealed cellular senescence landscape. *Nucleic Acids Research*, *50*(19), 10947–10963. 36243980 10.1093/nar/gkac885 PMC9638896

[58] Anderson, N. M., & Simon, M. C. (2020). The tumor microenvironment. *Current Biology*, *30*, R921–R925. 32810447 10.1016/j.cub.2020.06.081 PMC8194051

[59] Khanmammadova, N., Islam, S., Sharma, P., & Amit, M. (2023). Neuro-immune interactions and immuno-oncology. *Trends Cancer*, *9*, 636–649. 37258398 10.1016/j.trecan.2023.05.002 PMC10524972

[60] Yoneda,T., Hiasa, M., Okui, T., & Hata, K. (2021). Sensory nerves in bone metastasis. *J Bone Oncol*, *30*, 100387. 10.1016/j.jbo.2021.100387 34504741 PMC8411232

[61] Wu,C., Gong, S., Duan, Y., Deng, C., Kallendrusch, S., & Berninghausen, L. (2023). Tumor microenvironment-based prognostic index for osteosarcoma. *Journal Of Biomedical Science*, *30*(1), 917. 10.1186/s12929-023-00917-3 PMC10099847 37055822

[62] Zhi, X., Wu, F., Qian, J., Ochiai, Y., Lian, G., Malagola, E., et al. (2025). Nociceptive neurons promote gastric tumour progression via CGRP–RAMP1. *Nature*, *640*(8059), 802–810. 39972142 10.1038/s41586-025-08591-1 PMC13022952

[63] Gao, X., Zhang, D., Xu, C., Li, H., Caron, K. M., & Frenette, P. S. (2021). Nociceptive nerves regulate Haematopoietic stem cell mobilization. *Nature*, *589*(7843), 591–596. 33361809 10.1038/s41586-020-03057-y PMC7856173

[64] Kulalert, W., Wells, A. C., Link, V. M., Lim, A. I., Bouladoux, N., Nagai, M. (2024). Neuroimmune CGRP–RAMP1 axis in cutaneous immunity. *Proc Natl Acad Sci USA*. 121(11) :2322574121. 10.1073/pnas.2322574121 PMC10945812 38451947

[65] Balood, M., Ahmadi, M., Eichwald, T., Ahmadi, A., Majdoubi, A., Roversi, K., et al. (2022). Nociceptor neurons affect cancer immunosurveillance. *Nature*, *611*(7935), 405–412. 36323780 10.1038/s41586-022-05374-w PMC9646485

[66] Huang, S., Zhu, J., Yu, L., Huang, Y., & Hu, Y. (2025). Cancer–nervous system crosstalk. *Molecular Cancer*, *24*(1), 133. 40320550 10.1186/s12943-025-02336-4 PMC12051345

[67] Barbir, E. B., Kitchlu, A., & Herrmann, S. M. (2024). Immune checkpoint inhibitor-associated nephritis. *Nephrology, Dialysis, Transplantation*, *39*(11), 1785–1798. 39138117 10.1093/ndt/gfae184

[68] Patra, A. K. (2021). New aspects of HSC mobilization. *Cellular & Molecular Immunology*, *18*(12), 2583–2585. 34158632 10.1038/s41423-021-00723-7 PMC8632966

[69] Li,M., Li, L., Zheng, J., Li, Z., Li, S., & Wang, K. (2023). Liquid biopsy in renal cell carcinoma. *Molecular Cancer*, *22*, 1745. 10.1186/s12943-023-01745-7 PMC9942319 36810071

[70] Dallmayer,M., Li, J., Ohmura, S., Rubio, A., Baldauf, R., & Hölting, M. C., T. L. B (2019). Targeting the CALCB/RAMP1 axis inhibits ewing sarcoma growth. *Cell Death And Disease*, *10*(2), 1372. 10.1038/s41419-019-1372-0 PMC6370763 30741933

[71] Olivier, M., Asmis, R., Hawkins, G. A., Howard, T. D., & Cox, L. A. (2019). The need for multi-omics biomarker signatures. *International Journal of Molecular Sciences*, *20*(19), 4781. 31561483 10.3390/ijms20194781 PMC6801754

[72] Acharya, D., & Mukhopadhyay, A. (2024). Machine learning techniques for multi-omics data integration. *Briefings in Functional Genomics*, *23*(5), 549–560. 38600757 10.1093/bfgp/elae013

[73] Pekayvaz, K., Losert, C., Knottenberg, V., Gold, C., van Blokland, I. V., Oelen, R., et al. (2024). Multiomic analyses uncover immunological signatures. *Nature Medicine*, *30*(6), 1696–1710. 38773340 10.1038/s41591-024-02953-4 PMC11186793

[74] Yan,Z., Liu, Q., Cao, Z., Wang, J., Zhang, H., & Liu, J. (2022). Multi-omics integration reveals prognostic gene signature in neuroblastoma. *Front Neuroinform*, *16*, 1034793. 10.3389/fninf.2022.1034793 36439943 PMC9687102

[75] Liu, Z., Gao, Y., Zhang, W., Li, L., Ding, H., Zhang, S., et al. (2025). Regulatory mechanisms of paraptosis-related genes in gastric cancer. *Scientific Reports*, *15*(1), 36098. 41094022 10.1038/s41598-025-20037-2 PMC12528732

[76] Southekal, S., Shakyawar, S. K., Bajpai, P., Elkholy, A., Manne, U., Mishra, N. K., et al. (2023). Molecular subtyping of osteosarcoma. *Cancers (Basel)*, *15*(7), 2134. 37046795 10.3390/cancers15072134 PMC10093233

[77] Li, S., Chen, X., Chen, J., Wu, B., Liu, J., Guo, Y., et al. (2023). Multi-omics integration analysis of GPCRs in pan-cancer. *Computers in Biology and Medicine*, *161*, 106988. 37201441 10.1016/j.compbiomed.2023.106988

[78] Saliba, A., Du, Y., Feng, T., & Garmire, L. (2024). Multi-omics integration in nephrology. *Seminars in Nephrology*, *44*(6), 151584. 40216576 10.1016/j.semnephrol.2025.151584 PMC12278776

[79] Dong, R., Chen, S., Lu, F., Zheng, N., Peng, G., Li, Y. (2022). Models predicting immunotherapy response in gastric cancer. *Biomed Res Int*. 2022.

[80] Liu, G., Tian, X., Wang, Q., Xu, S., Jiang, Y., Gao, Y., et al. (2025). Prdm12 links neuroimmune crosstalk to CD8 + T cell exhaustion. *Science Advances*, *11*, 33. 10.1126/sciadv.adx9221 PMC12356268 40815657

[81] Lorente, J. S., Sokolov, A. V., Ferguson, G., Schiöth, H. B., Hauser, A. S., & Gloriam, D. E. (2025). GPCR drug discovery. *Nature Reviews. Drug Discovery*, *24*(6), 458–479. 40033110 10.1038/s41573-025-01139-y

[82] Cornwell, A. C., & Feigin, M. E. (2020). Unintended effects of GPCR-targeted drugs on cancer phenotype. *Trends in Pharmacological Sciences*, *41*(12), 1006–1022. 33198923 10.1016/j.tips.2020.10.001 PMC7672258

[83] Edvinsson, L., Haanes, K. A., Warfvinge, K., & Krause, D. N. (2018). CGRP as the target of new migraine therapies. *Nat Rev Neurol*, *14*(6), 338–350. 29691490 10.1038/s41582-018-0003-1

